# Asymmetric Double Freeform Surface Lens for Integrated LED Automobile Headlamp

**DOI:** 10.3390/mi12060663

**Published:** 2021-06-05

**Authors:** Hui Zhang, Dengfei Liu, Yinwan Wei, Hong Wang

**Affiliations:** 1 Engineering Research Center for Optoelectronics of Guangzhou Province, School of Physics and Optoelectronics, South China University of Technology, Guangzhou 510640, China; 201820127932@mail.scut.edu.cn (H.Z.); eedfliu@mail.scut.edu.cn (D.L.); 202020130343@mail.scut.edu.cn (Y.W.); 2 Guangdong Provincial Engineering Research Center for Wide Bandgap Semiconductor Chips and Application, Zhongshan Institute of Modern Industrial Technology, South China University of Technology, Zhongshan 528437, China

**Keywords:** asymmetric double freeform surface lens, LED, integrated automobile headlamp, freeform surface optics

## Abstract

We propose a design method of asymmetric double freeform surface lens for an integrated LED automobile headlamp and develop an integrated LED automobile optical system. A single asymmetric double freeform surface lens is designed to redistribute rays emitting from the light source for realizing both low and high beams. Moreover, a freeform surface reflector is used to improve the energy efficiency of high beams. The prism placed in the optical path can suppress chromatic dispersion on the edge of the target plane. Simulation and experimental results show that the illumination values and color temperature of the key points can fully meet the requirements of United Nations Economic Commission for Europe vehicle regulations (ECE) R112, 48, and 128. The volume of the whole optical system comprised of freeform surface elements is smaller than that of the low beam system of a traditional headlamp, resulting in saved space, in which other electronic devices can be installed for the safety of the driver, which indicates that the proposed method is practical in the field of automobile lighting.

## 1. Introduction

LEDs have gradually entered our lives due to their advantages of energy saving, high energy efficiency, long lifespan, and miniaturization [1,2]. In the past decade, LEDs have been used increasingly as light sources in automobile headlamps for their excellent properties [3,4,5,6]. Since low-beam requirements are completely different from those of high beams, the traditional optical systems of low and high beams in headlamps are designed separately, mainly including projection and reflection types. In general, projection-type optical systems deflect by lenses and reflection type by reflectors [7]. It is easy to form a clear cutoff line but hard to improve the energy efficiency for projection types with a baffle plate; conversely, the reflection type without a baffle plate is more efficient but has a dim cutoff line. Previously, Cvetkovic et al. presented a simultaneous multiple surface (SMS) 3D method for automobile low and high beam headlamps, whose optical efficiency is more than 75% [8,9]. Domhardt et al. designed a combined lens for LED-based low beam headlamp but needed large space to install lenses for high energy efficiency [10]. Ge et al. used an elliptical and parabolic reflector to design a low beam headlamp that had high energy efficiency [5]. Hsieh et al. proposed a modular design for an LED vehicle projector headlamp that provided the cutoff line of low beams without a baffle plate [11]. Chu et al. proposed a low beam headlamp with a compound ellipsoidal reflector that achieved the highest energy efficiency in the existing literature [12]. In the past, our research group has proposed several optical systems for headlamps. For example, an optical system with a single freeform surface lens [13], a low chromatic dispersion headlamp system using a parabolic reflector and micro-lens array [14], a LED motorcycle headlight using a combined lens [15], etc. More separate design methods for low and high beams can be found in references [16,17,18,19,20,21]. In recent years, compact headlamp design has become more and more fashionable [22]. The integrated headlamp reduces its volume by sharing some optical elements between low- and high-beam systems, allowing the designs of integrated headlamps to fit with the trend of compact design. However, few studies in the literature have been reported about the integrated headlamp. Hung et al. designed an integrated headlamp incorporating a digital micromirror device [23]. Wu et al. presented a modular LED headlamp system based on a freeform reflector [24]. Borocki et al. recommended a single optical system with both a low beam and a high beam, in which switching between the low beam and high beam was achieved by a removable shutter [25]. M. Rice et al. proposed an integrated headlamp based on a projective low-beam system, in which the light emitted from the high-beam source was reflected by the lower plane of the baffle plate and deflected by the lens to form the illumination distribution of high beams [26]. In previous studies, most of the headlamps were designed individually, which may increase the complexity and volume of the headlamp. Existing integrated headlamps with a removable shutter may affect the reliability of the headlamp. Therefore, it is necessary to study a simple and small-volume headlamp system.

In this paper, we proposed a method of designing an asymmetric double freeform surface lens (ADFSL) and developed an integrated LED automobile optical system. On the premise of meeting the requirements of ECE R112, 48, and 128 [27,28,29], this new integrated LED headlamp system can greatly improve the compactness of the optical system and suppress chromatic dispersion at the edge of the target illumination area. Just one lens is needed to achieve low and high beams, which means that this new headlamp system can save a lot of space. A freeform surface reflector focusing light is proposed to improve energy efficiency. Furthermore, a prism is placed in the optical path to reduce the chromatic dispersion further, which can provide comfortable and safe lighting for the driver. Compared to the traditional, separated headlamp and integrated headlamp, the proposed headlamp has only one single lens to achieve low and high beams, reducing the volume of the automobile headlamp. Furthermore, the color distribution on the target plane using ADFSL and prism is more stable.

## 2. Design Method

**Figure 1:**
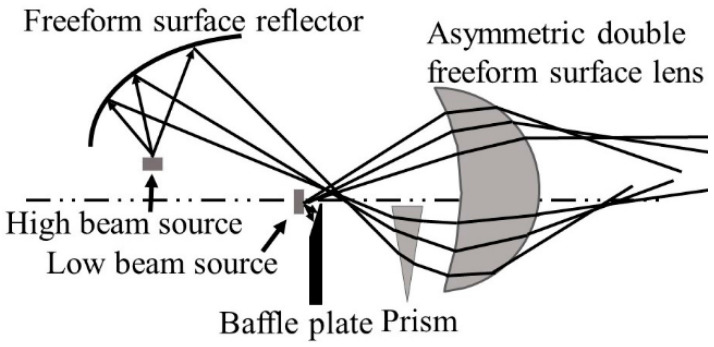
Optical system of the integrated LED automobile headlamp.

The optical system of the new integrated headlamp is shown in Figure 1. The whole system consists of an ADFSL, a freeform surface reflector, a prism, a baffle, and two LEDs. The optical system of the low beam includes a LED source, an upper half of the ADFSL, and a baffle plate. Correspondingly, the optical system of the high beam comprises a freeform surface reflector, a prism, the lower half of the ADFSL, and a LED source. Rays emitting from the low beam LED source are partially blocked by the baffle plate and then redistributed by the ADFSL, forming a low beam with a clear cutoff line on the target plane. On the other hand, rays from the high beam LED source are focused on a point by the freeform surface reflector and then deflected by the prism. Finally, the remaining rays will be redistributed by the ADFSL to form the illumination distribution of the high beam. The implementation of the low and high beams will be described in detail below.

### 2.1. Optical System of the Low Beam Mode

**Figure 2:**
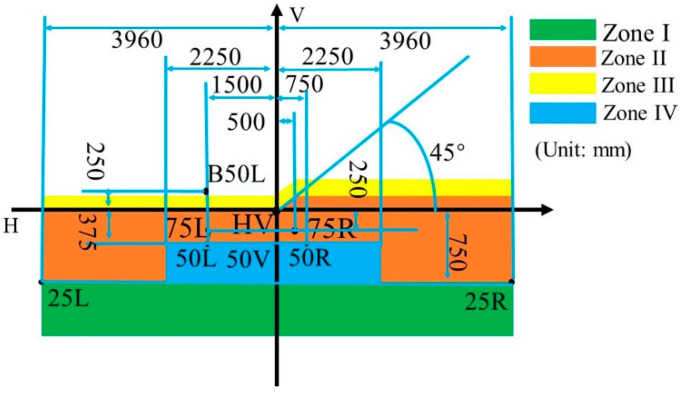
Tested points and regions of low beam.

Over the past decade, a series of regulations have been introduced for driver’s safety. Low beam is mainly used for good road lighting and cannot cause glare to the opposing drivers. According to the ECE R112 regulations, the tested points and the cutoff line are shown in Figure 2.

**Figure 3:**
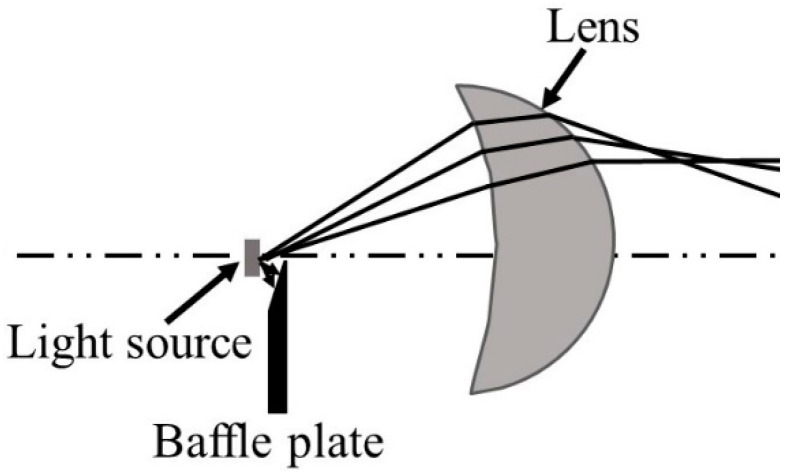
Low beam system without an ellipsoid reflector.

It can be found that the light shape of the low beam has a wide horizontal spread for both sides, which can provide a big angle of view for drivers. Moreover, the lighting spot of the low beam is “dark on the left but bright on the right”. The measuring plane is 25 m in front of the headlamp for testing the illuminances on key points. Each optical element has a specific function to ensure a qualified low beam on the measuring plane. Concretely, the upper half of the ADFSL is used to realize the ellipse lighting spot, and the baffle plate blocks the stray light to form a clear cutoff line. In our laboratory, a low beam system for laser headlamp without ellipsoid reflector (Figure 3) was designed, and the construction was also adopted in the integrated headlamp for the low beam for the compactness of the optical system.

**Figure 4:**
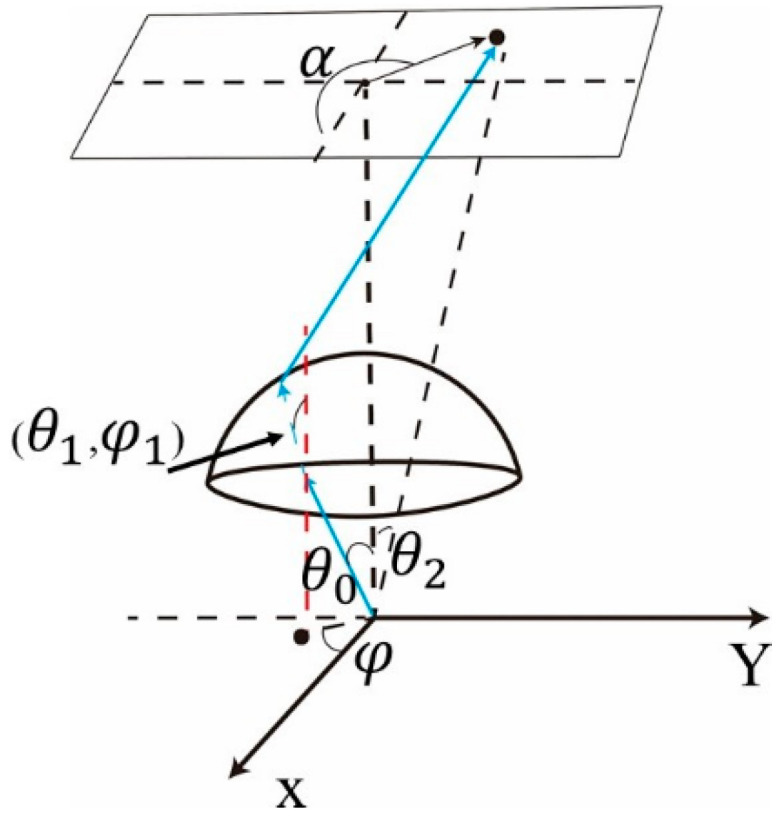
Sketch of the light deflection of the asymmetric double freeform surface lens.

Double freeform surface lens (DFSL) has many advantages over single freeform surface lens. For example, it can achieve an accurate distribution of light and suppress chromatic dispersion by its inner and outer freeform surfaces [15]. The deflection factor C (0 ≤ C ≤ 1), an important factor in DFSL, is used to represent the angular deflection ratio of two freeform surfaces. It is worth noting that θ, φ are zenith angle and azimuth angle, respectively, and α is the angle between the discrete points on the target plane and positive X axis (Figure 4). The ray’s angle after passing through the inner freeform surface could be denoted as follows:

(1) $$\begin{array}{l} \theta_{1}=C\theta_{0}+(1-C)\theta_{2}\\ \varphi_{1}=C\varphi +(1-C)\alpha \end{array}$$

**Figure 5:**
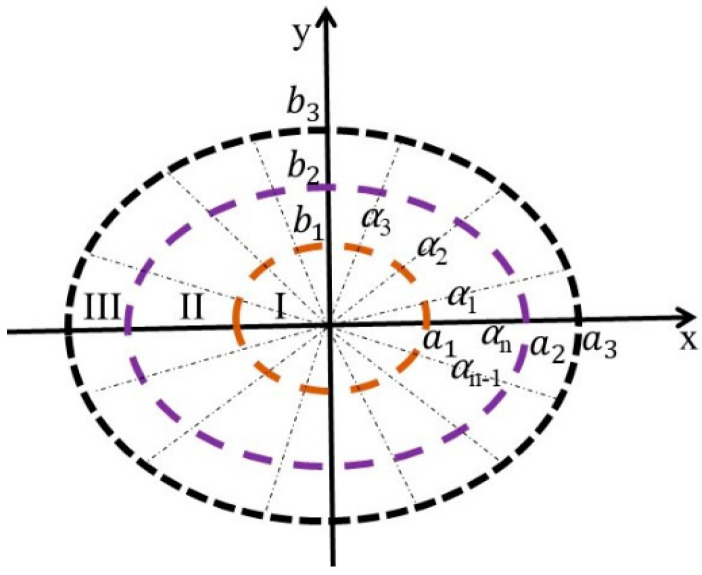
Division of the target plane for low beam.

To construct the ADFSL, the mapping relationship between certain angle rays on the target plane and discrete points of the target plane should be calculated. Figure 5 shows the division of the target illumination area. To simplify the calculation and generate qualified illumination distribution, the lighting zone is set as an ellipse and divided into I, II, III zones. *E1*, *E2*, *E3* are the corresponding illuminances of zone I, II, III, respectively, and the semi-major axis is *a*, and the semi-minor axis, *b*. Similarly, the semi-major and semi-minor axes are equally divided into three parts, with $a_{i}$, $b_{i}$ being the semi-major axis and semi-minor axis of the divided sub-ellipse, respectively. On the other hand, the target illumination area is divided into *n* parts along the direction α, where $\alpha_{i}$ is the angle between *i*th part and the positive *X* axis.

$I_{0}$ is the central intensity of the LED source; $I_{\left(\theta \right)}$, the intensity distribution of light in the θ direction; θ, the angle between the emergent light and the optical axis; and the relationship between $I_{\left(\theta \right)}$ and $I_{0}$ can be expressed as follows:

(2) $$I_{\left(\theta \right)}=I_{0}\cos\theta$$

According to the conservation of energy, we obtain [30]

(3) $$\begin{array}{l} 2\pi \int_{0}^{\theta_{1}}I_{0}\cdot \cos\theta \cdot \sin\theta d\theta =\frac{1}{2}\int_{0}^{\alpha_{n}}E1\cdot \frac{a_{1}^{2}b_{1}^{2}}{b_{1}\cos^{2}\alpha +a_{1}\sin^{2}\alpha }d\alpha \\ 2\pi \int_{\theta_{1}}^{\theta_{2}}I_{0}\cdot \cos\theta \cdot \sin\theta d\theta =\frac{1}{2}\int_{0}^{\alpha_{n}}E2\cdot \left(\frac{a_{2}^{2}b_{2}^{2}}{b_{2}\cos^{2}\alpha +a_{2}\sin^{2}\alpha }-\frac{a_{1}^{2}b_{1}^{2}}{b_{1}\cos^{2}\alpha +a_{1}\sin^{2}\alpha }\right)d\alpha \\ 2\pi \int_{\theta 2}^{\theta_{3}}I_{0}\cdot \cos\theta \cdot \sin\theta d\theta =\frac{1}{2}\int_{0}^{\alpha_{n}}E3\cdot \left(\frac{a_{3}^{2}b_{3}^{2}}{b_{3}\cos^{2}\alpha +a_{3}\sin^{2}\alpha }-\frac{a_{2}^{2}b_{2}^{2}}{b_{2}\cos^{2}\alpha +a_{2}\sin^{2}\alpha }\right)d\alpha \end{array}$$

By solving the above integral equation, the mapping relationship between $\theta_{i}$ and the illumination area on the target plane can be acquired.

According to the conservation of energy, the relationship between the azimuth angle $\varphi_{j}$ and $\alpha_{j}$ can be expressed as follows:

(4) $$\begin{array}{l} \int_{0}^{\theta_{1}}\int_{\varphi_{j}}^{\varphi_{j+1}}I_{0}\cdot \cos\theta \cdot \sin\theta d\theta d\varphi =\frac{1}{2}\int_{\alpha_{j}}^{\alpha_{j+1}}E1\cdot \frac{a_{1}^{2}b_{1}^{2}}{b_{1}\cos^{2}\alpha +a_{1}\sin^{2}\alpha }d\alpha \\ \int_{\theta_{1}}^{\theta_{2}}\int_{\varphi_{j}}^{\varphi_{j+1}}I_{0}\cdot \cos\theta \cdot \sin\theta d\theta d\varphi =\frac{1}{2}\int_{\alpha_{j}}^{\alpha_{j+1}}E2\cdot \left(\frac{a_{2}^{2}b_{2}^{2}}{b_{2}\cos^{2}\alpha +a_{2}\sin^{2}\alpha }-\frac{a_{1}^{2}b_{1}^{2}}{b_{1}\cos^{2}\alpha +a_{1}\sin^{2}\alpha }\right)d\alpha \\ \int_{\theta_{2}}^{\theta 3}\int_{\varphi_{j}}^{\varphi_{j+1}}I_{0}\cdot \cos\theta \cdot \sin\theta d\theta d\varphi =\frac{1}{2}\int_{\alpha_{j}}^{\alpha_{j+1}}E3\cdot \left(\frac{a_{3}^{2}b_{3}^{2}}{b_{3}\cos^{2}\alpha +a_{3}\sin^{2}\alpha }-\frac{a_{2}^{2}b_{2}^{2}}{b_{2}\cos^{2}\alpha +a_{2}\sin^{2}\alpha }\right)d\alpha \end{array}$$

By solving the above equation, the relationship between $\varphi_{j}$ and $\alpha_{j}$ is obtained.

(5) $$\left\{ \begin{array}{l} x_{(i,j)}=a_{i}\cos\alpha_{j}\\ y_{(i,j)}=b_{i}\sin\alpha_{j} \end{array}\right.$$

**Figure 6:**
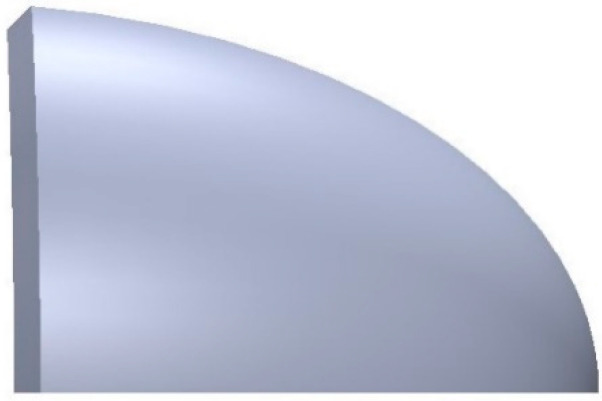
The upper half of the double freeform surface lens.

The coordinates of the points on the target plane can be calculated by Equation (5). Then, we obtain the mapping relationship between the light emitting from the source and the points on the target plane. Next, the profile of ADFL is calculated by iteration. The initial points of the first and second freeform surfaces are set as L1 and L2, respectively. After the initial points are given, the normal vector of the initial points can be obtained according to the vector form of refraction law,

(6) $$\vec{N}=\frac{n_{o}\vec{out}-n_{in}\vec{In}}{\left|n_{o}\vec{out}-n_{in}\vec{In}\right|}$$

where $\vec{In}$ is the unit vector of the incident ray, $\vec{Out}$ is the unit vector of outgoing ray, $\vec{N}$ is the normal vector, $n_{o}$ is the refractive index in the outgoing space, and $n_{in}$ is the refractive index in the incident space. All coordinates of the discrete points on the freeform surface can be calculated by iteration of the normal vector, which stipulates that the intersection point of the next incident ray and the normal plane of the last point is the next point on the curve. The expression of the normal plane at any point can be obtained by Equation (6). By fixing the value of φ and changing the value of θ, the initial curve of the first and second freeform surfaces is generated. Then, by fixing the value of θ and changing the value of φ, the coordinates of the discrete points on both freeform surfaces can be acquired by iteration of the normal vector. Readers can find the detailed iteration process in [15]. After importing the data to modeling software, the upper half of the ADFSL can be obtained by lofting curve, as shown in Figure 6.

**Figure 7:**
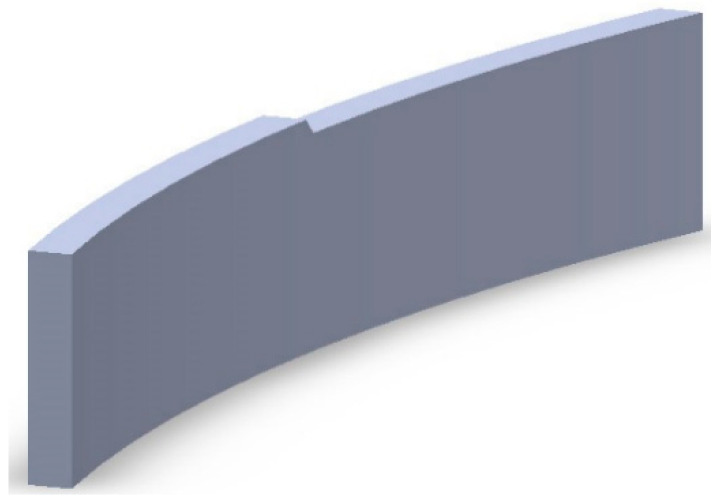
The baffle plate.

The curved baffle plate with 45 degrees is chosen to form the clear cutoff line, as shown in Figure 7.

### 2.2. Optical System of the High Beam Mode

**Figure 8:**
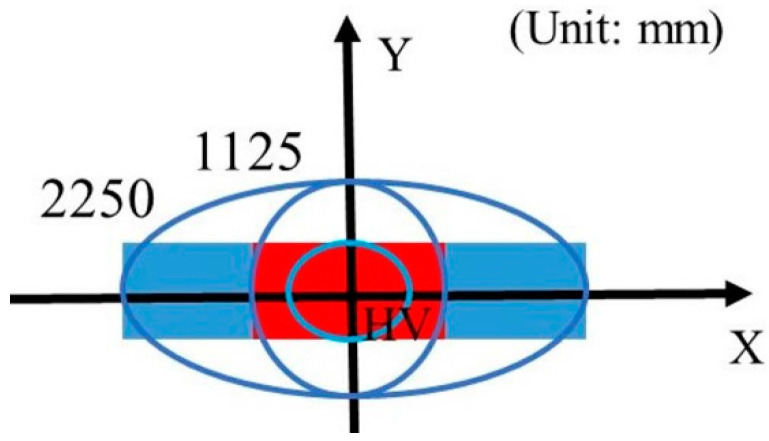
Test points and regions of the high beam.

The high beam of the automobile headlamp is mainly used for bad road lighting since it provides enough illumination on the target area [31]. In addition, the regulations require the target plane center area with maximum illumination. The test area of the high beam is shown in Figure 8.

**Figure 9:**
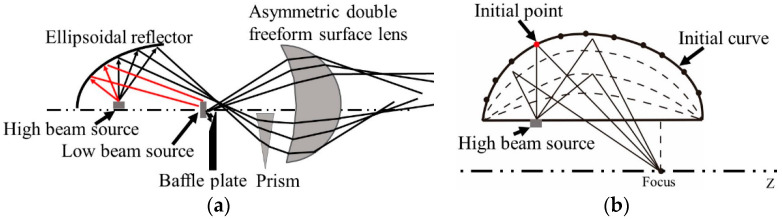
(**a**) Integrated headlamp based on ellipsoidal reflector and (**b**) sketch of the freeform reflector.

The traditional design uses an ellipsoidal reflector to focus the rays emitted from the source, while part of the rays emitted from the high beam source will be blocked by the low beam source due to the volume of the LED source. It will cause significant energy losses, as shown in Figure 9a. Therefore, the construction in Figure 1 is adopted to improve light efficiency and illumination. Rays emitted from the high beam source focus on a point on Z axis by the freeform surface reflector, as shown in Figure 9b.

Due to the symmetry of the reflector, only half of the freeform surface reflector was designed. An initial point is given and the normal vector of the initial point can be obtained according to the vector form of the reflection law, which can be expressed as

(7) $$\vec{N}=\frac{\vec{out}-\vec{In}}{\left|\vec{out}-\vec{In}\right|}$$

where $\vec{In}$, $\vec{Out}$ are the unit vectors of the incident ray and outgoing ray, respectively, and $\vec{N}$ is the normal vector. The initial curve and profile of the freeform surface reflector can be obtained by an iteration process.

**Figure 10:**
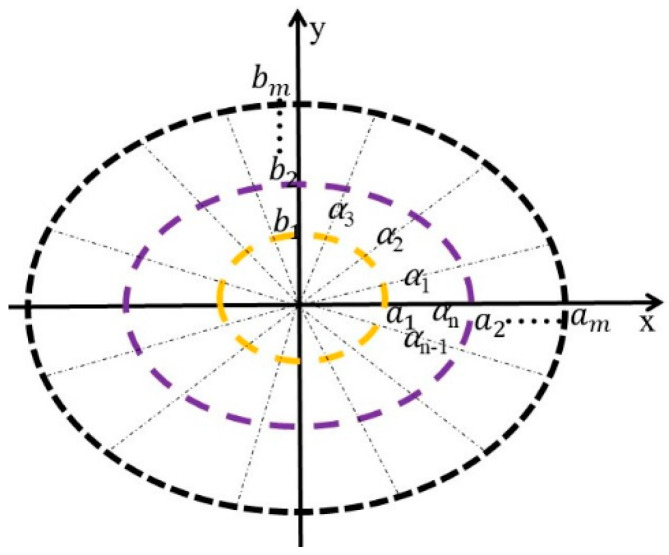
Division of the target plane for high beam.

Freeform surface reflector converges rays emitted from the source to another focus on the *Z* axis. Therefore, the focus on the *Z* axis can be seen as a light source without volume that does not block the rays from the low beam source and will illuminate the target plane. Similar to the process of low beam design, the lighting spot of the high beam is set as an ellipse, whose semi-major axis and semi-minor axis are equally divided into *m* parts. Accordingly, $a_{i}$, $b_{i}$ are the semi-major and semi-minor axes of *i*th part, respectively, 0 ≤ *i* ≤ m. At the same time, the target plane is also divided into *n* parts along the α direction, $\alpha_{i}$ is the angle between *i*th part and the X axis, and then the target plane is divided into m×n lattices, as shown in Figure 10. According to the energy conservation, we can obtain the mapping relations.

(8) $$\int_{\theta_{i}}^{\theta_{i+1}}\int_{\varphi_{j}}^{\varphi_{j+1}}I_{\left(\theta ,\varphi \right)}d\theta d\varphi =\frac{1}{2}\int_{\alpha_{j}}^{\alpha_{j+1}}E_{0}\cdot k_{i}\cdot \left(\frac{a_{i+1}^{2}b_{i+1}^{2}}{b_{i+1}\cos^{2}\alpha +a_{i+1}\sin^{2}\alpha }-\frac{a_{i}^{2}b_{i}^{2}}{b_{i}\cos^{2}\alpha +a_{i}\sin^{2}\alpha }\right)d\alpha$$

where *E*_0_*∙k_i_* is the illumination value, and *k_i_* is the illuminance control factor on the *i*th part. Generally, the closer to the original point, the greater the value of the *k_i_*. I(θ,φ) is the intensity distribution of the light focused by the freeform surface reflector. After calculating Equation (8), the mapping relationship can be acquired, and the profile of the lower half of the ADFSL is calculated by the normal vector iteration.

**Figure 11:**
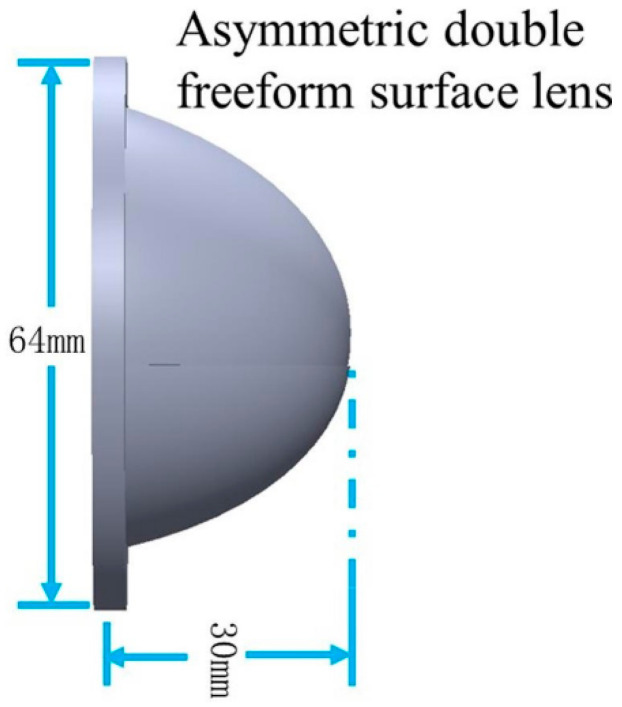
The asymmetric double freeform surface lens.

Similar to the low beam lens construction method, the whole ADFSL can be built in the modeling software. As shown in Figure 11, the diameter and thickness of the lens are 64 mm and 30 mm, respectively.

(9) $$\sin(\frac{\alpha +\delta }{2})=n\sin(\frac{\alpha }{2})\cdot \frac{\cos\frac{{I^{\prime }}_{1}+I_{2}}{2}}{\cos\frac{I_{1}+{I^{\prime }}_{2}}{2}}$$

**Figure 12:**
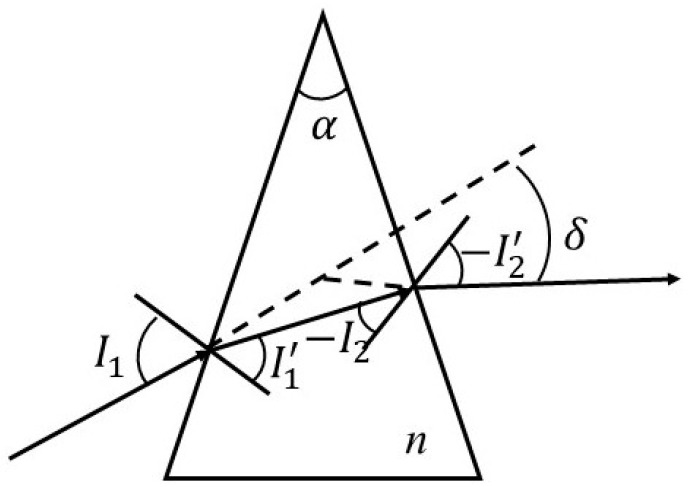
Optical path in the prism.

Although DFSL can reduce chromatic dispersion on the target plane, there is still serious chromatic dispersion at the edge due to the large deflection angle. To obtain a light pattern with a stable color distribution, a prism was added to the optical system to reduce the deflection angle of the ADFSL. Figure 12 shows an optical path in the prism. The prism can deflect rays based on refraction law, and the deflection angle between the incident and outgoing ray is δ. Formula (9) presents the calculation of the deflection angle of the prism [32].

## 3. Simulation

**Figure 13:**
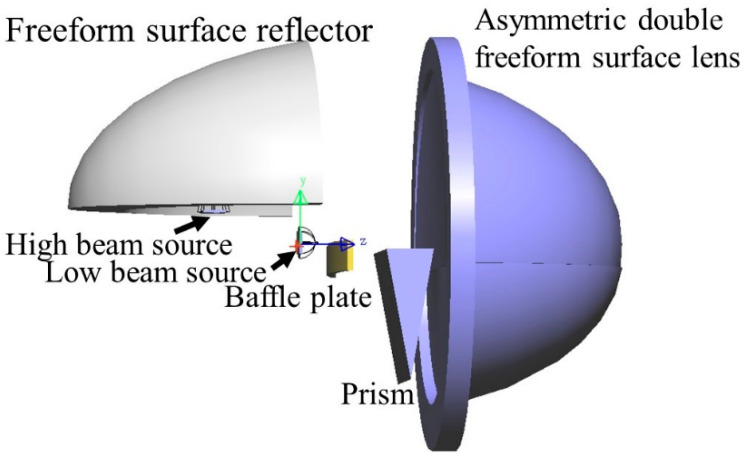
Optical system of the integrated headlamp in simulation software.

After calculation and optimization, the whole system of the integrated headlamp is introduced into the optical simulation software LightTools [33], as shown in Figure 13. The volume of the whole system is 91 × 64 × 64 mm^3^, which is smaller than all the separately designed headlamp systems. The low beam source placed at the origin is KW H4L531.TE of OSRAM company and 6R brightness with 1300 lm luminous flux is selected [34]. On the other hand, the high beam source placed at a focus of the freeform surface reflector is KW H4L531.TE and 7R brightness with 1500 lm luminous flux is selected. Low and high beam sources comply with the ECE R48 and ECE R128 regulations [28,29].

Before the simulation, the freeform surface reflector is set as an aluminum alloy with a reflectivity of 90% and the lens material is PMMA with a refractive index of 1.49. The baffle plate is set as a perfect absorber to absorb all rays that hit it. The exact illuminance values on key points and the specified regions in the prescribed area can be read in the software. The simulation results are shown in the figures and tables below.

**Figure 14:**
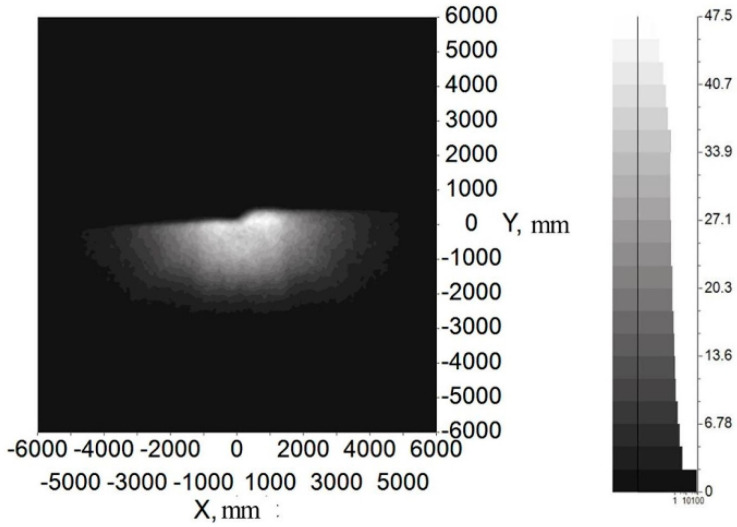
The illumination distribution of low beam.

**Figure 15:**
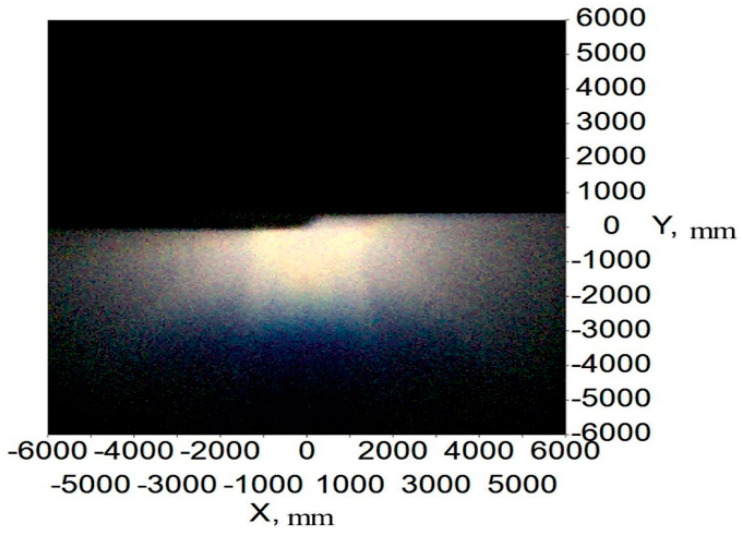
The color distribution of low beam.

**Figure 16:**
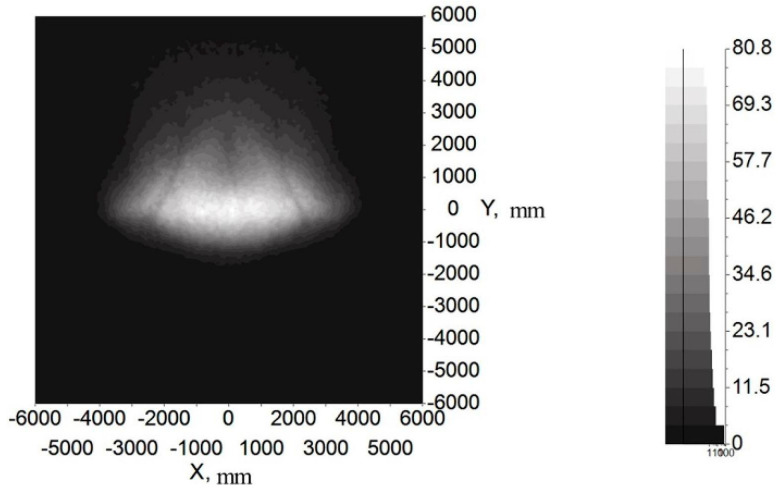
The illumination distribution of high beam with prism.

**Figure 17:**
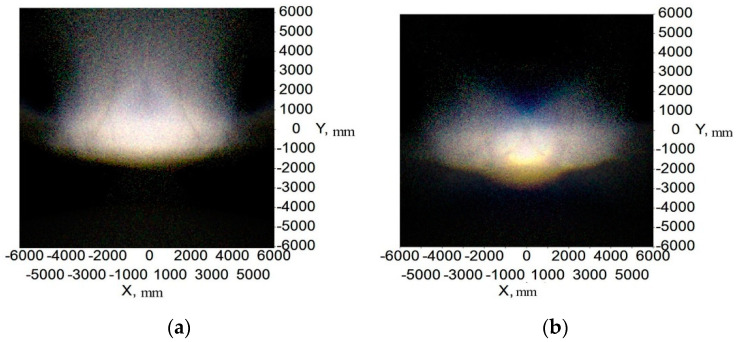
(**a**) The color distribution of high beam with a prism and (**b**) without a prism.

Figure 14 and Figure 15 show the illumination and color distribution of the low beam, respectively. Figure 16 shows the illumination distribution of the high beam. Figure 17a shows the color distribution of a high beam with a prism, and Figure 17b shows the color distribution without a prism. Table 1 and Table 2 show the simulated illuminance values on the target plane of low and high beams, respectively.

Figure 14 and Figure 15 indicate that the integrated headlamp system with a single ADFSL could ensure the quality of the low beam. The illumination width of the low beam can reach 12 m, which can provide a large angle of view for drivers. Furthermore, the cutoff line with the blue edge does not appear, which means that the inherent problem of the cutoff line with the blue edge in traditional projection designs can be solved by this method. The color temperature of the low beam is stable on the target plane, and the color on the target plane is close to white. As shown in Table 1, the simulation values of the low beam fully meet the requirements of ECE R112 regulations; Figure 16 shows that the illumination distribution of the high beam can comply with the requirements of ECE R112 regulations. Furthermore, the highest value of the high beam is 80.8 lux, providing sufficient illumination when the road lighting is poor. Figure 17 shows the color distribution of the high beam. The color distribution with a prism is more stable, as compared to the color distribution without a prism, which means that adding a prism to the optical path can largely suppress chromatic dispersion on the edge. Table 2 demonstrates that the simulation values of the high beam satisfy the requirements of ECE R112 regulations. In short, the light shape of the low beam and high beam and simulation values of the key points fully comply with the requirements of ECE R112; Moreover, the chromatic dispersion can be largely suppressed by using a new integrated optical system.

Here, we must present a simple analysis of the installation error. The error of the low beam and high beam source position mounting is ±0.3 mm and 0.5 mm, respectively. In other words, when position error is greater than the above values, the performance will no longer satisfy the ECE R112 regulations. Therefore, proper installment of the optical elements is very important for achieving optimal performance.

## 4. Experiment

**Figure 18:**
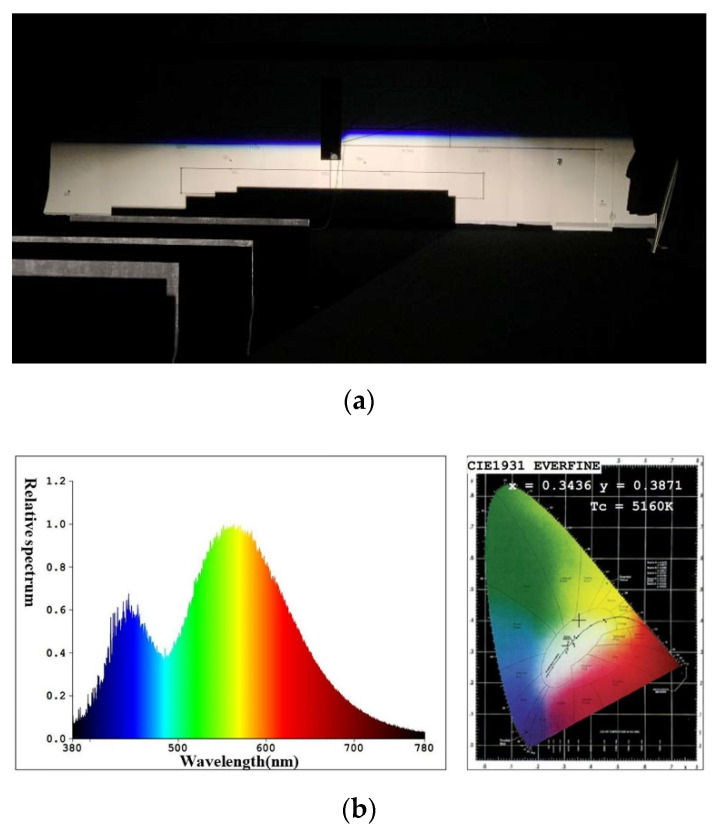
(**a**) The lighting spot and (**b**) test results of color of the point 50 V for low beam.

**Figure 19:**
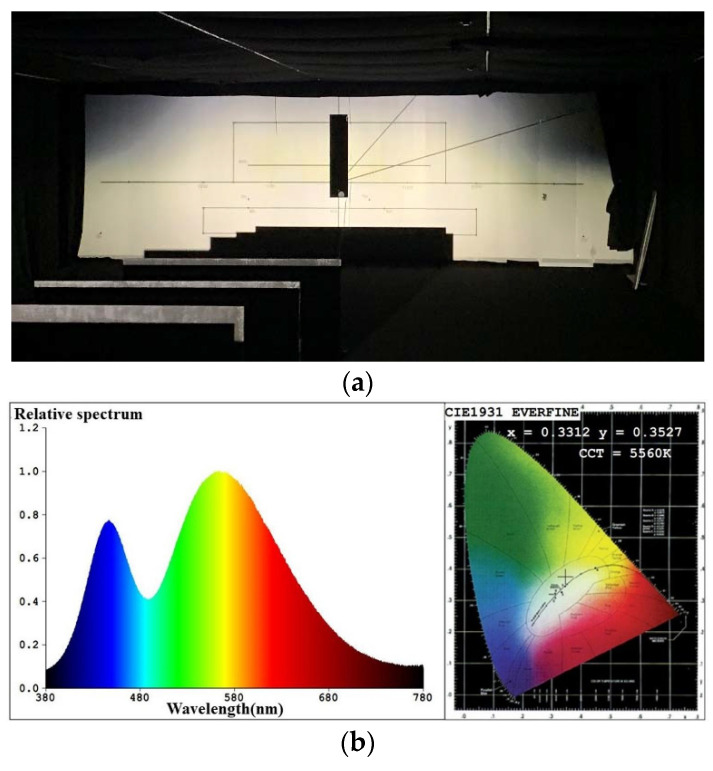
(**a**) The lighting spot and (**b**) test results of color of the point HV for high beam.

To study the illumination performance of the experiment, the optical samples were processed and assembled according to the results of the optical simulation software, and the GO-HD5 automobile lighting test system was used for testing the illuminance values of the key points and specified regions. According to ECE R112 regulations, the test starts after the light source is steady. The lighting spot of the low beam and color temperature test of the point 50 V are shown in Figure 18, and the lighting spot of the high beam and color temperature test of the point HV are shown in Figure 19. The test illumination values of the low beam and high beam measurement screens are shown in Table 3 and Table 4, respectively.

From Figure 18a, there is a straight cutoff line with no stray light above, and the color temperature is stable on the measuring screen. Slight chromatic dispersion appears near the cutoff line, which is different from the simulation results. Additionally, it still meets the ECE regulations. Compared with the traditional low beam, the chromatic dispersion near the cutoff line is much slighter. The test results of illuminance were slightly different from the simulations, due to the slight error of the shield’s place and the absorption of mechanical objects. However, all illuminance values at the required points can fit with the regulations. According to ECE regulations, the color test results for the 50 V point must be within the white light range. The color test result at point 50 V is 5160 K, as shown in Figure 18b, and is quite qualified. The low beam lighting spot has a smooth transition that provides drivers with visual comfort.

It can be inferred from Figure 19a and Table 4 that the lighting spot is nearly elliptic and the central area is the brightest, which meets the ECE regulations. The color of the high beam is almost white, indicating that adding a prism could reduce chromatic dispersion at the edge of the target area and stabilize the color temperature. Similar to the test low beam results, the high beam test results in Table 4 are lower than the simulation results. The maximum illuminance of the high beam is 70.36 lux, less than simulation results but still much greater than the regulation requires, and can provide sufficient illumination for the ground. Similarly, the color test results for the HV point must be within the white light range. The color test result of the HV point is 5560 K, as shown in Figure 19b, which complies with the regulations.

## 5. Discussion

According to the simulation and experimental results, it can be found that both illuminance values of the required points and the color temperature distributions are qualified. The comparison of the new integrated headlamp system with the traditional headlamp system is shown in Table 5.

The volume of the new integrated LED headlamp is 91 × 64 × 64 mm^3^, much smaller than that of the traditional headlamp. The ADFSL has a diameter of 64 mm, as large as the traditional low beam lens, which means saving space for installing the high beam system and its cooling system. Compared with the high beam without prism, the new headlamp with ADFSL and prism has a more uniform color distribution due to the light deflection of the prim. Both low and high beams are qualified. Since the new integrated headlamp has only one optical system, it costs less than the traditional headlamp with two optical systems. By using an asymmetric double freeform surface lens, the cutoff line of the new integrated headlamp has less chromatic dispersion than that of the traditional headlamp using a single freeform surface or aspheric surface lens, providing comfortable lighting for oncoming drivers. These advantages make the new integrated headlights acceptable in the headlamp market.

## 6. Conclusions

We proposed a method to design the asymmetric double freeform surface lens and the integrated LED headlamp optical system. The construction algorithms of the optical elements were presented in detail. Simulation and experimental results show that a good light pattern can be obtained with the selected source, and the values of key points and specified regions can fully comply with the standards of ECE R112, 48, and 128. Moreover, color uniformity on the target plane is improved by the freeform surface lens and the prism. The volume of the whole optical system is smaller than that of the traditional headlamp, saving space to install other electronic devices safely, such as distance sensors, to help drivers gain better control of the distances between vehicles. Moreover, the freeform surface optical system has high compactness and manufacturing feasibility.

## Figures and Tables

**Table 1:** Simulated illuminance values of low beam on the target plane.

Point on Target Plane	Required Illuminance in Lux	Simulated illuminance in Lux
HV	≤0.7	0.28
B50L	≤0.4	0.00
75L	≤12	6.50
50L	≤15	13.36
75R	≥15	41.50
50R	≥12	40.48
50V	≥6	35.08
25L	≥2	5.22
25R	≥2	5.67
Zone I	≤2E *	√
Zone III	≤0.7	√
Zone IV	≥3	√

E * is the actual measurement of the value at point 50R.

**Table 2:** Simulated illuminance values of high beam on the target plane.

Point on Target Plane	Required Illuminance in Lux	Simulated Illuminance in Lux
E_max_	≥48 and ≤240	80.80
HV	≥0.8 E_max_	74.81
0–1125L, 1125R	≥24	√
0–2250, 2250R	≥6	√

**Table 3:** Tested illuminance values of low beam on the target plane.

Point on Target Plane	Required Illuminance in Lux	Tested Illuminance in Lux
HV	≤0.7	0.42
B50L	≤0.4	0.00
75L	≤12	7.36
50L	≤15	13.25
75R	≥15	32.32
50R	≥12	30.26
50V	≥6	34.26
25L	≥2	6.32
25R	≥2	6.85
Zone I	≤2E *	√
Zone III	≤0.7	√
Zone IV	≥3	√

E * is the actual measurement of the value at point 50R.

**Table 4:** Tested illuminance values of high beam on the target plane.

Point on Target Plane	Required Illuminance in Lux	Tested Illuminance in Lux
E_max_	≥48 and ≤240	70.36
HV	≥0.8 E_max_	65.34
0–1125L, 1125R	≥24	√
0–2250L, 2250R	≥6	√

**Table 5:** The comparison of the new integrated headlamp with traditional headlamp.

Items	Volume	Cost	Low Beam	High Beam	Cutoff Line	Chromatic Dispersion on the Target Plane
New integrated headlamp system	Small	Low	Qualified	Qualified	Slight chromatic dispersion	Slight
Traditional projection headlamp system	Large	High	Qualified	Qualified	A little serious chromatic dispersion	A little serious

## Data Availability

The data presented in this study are included in the article.
